# Combination Therapy of Ursodeoxycholic Acid and Corticosteroids for Primary Biliary Cirrhosis with Features of Autoimmune Hepatitis: A Meta-Analysis

**DOI:** 10.1155/2013/490731

**Published:** 2013-12-04

**Authors:** Yan Zhang, Jie Lu, Weiqi Dai, Fan Wang, Miao Shen, Jing Yang, Rong Zhu, Huawei Zhang, Kan Chen, Ping Cheng, Lei He, Chengfen Wang, Ling Xu, Yingqun Zhou, Chuanyong Guo

**Affiliations:** Department of Gastroenterology, Shanghai Tenth People's Hospital, Tongji University School of Medicine, Shanghai 200072, China

## Abstract

A meta-analysis was performed of RCTs comparing therapies that combine UDCA and corticosteroids with UDCA monotherapy. In this paper, we found that the combination therapy of UDCA and corticosteroids was more effective for PBC-AIH.

## 1. Introduction

Primary biliary cirrhosis (PBC) and autoimmune hepatitis (AIH) are two autoimmune diseases that have major effects on the liver. Each disease has its own clinical manifestations and immunological and histological features [1]. However, some patients may display the characteristics of both diseases. For example, patients with PBC with features of AIH have the characteristics of both PBC and AIH simultaneously [2]. According to the study by Czaja [3], the incidence of PBC-AIH in autoimmune liver disease is 7%. Chazouillères et al. [4] concluded that the incidence of PBC-AIH in PBC is 9.2%. Because the incidence of PBC-AIH is low and there have been a few large-scale randomized controlled trials (RCTs) about it; its treatment is largely based on experience. Joshi et al. [5], research scientists, reported that when 16 patients with PBC-AIH were treated correctly with ursodeoxycholic acid (UDCA) (13–15 mg kg^−1^ per day), their survival rate did not differ from that of patients with PBC. Renou et al. [6], other research scientists, reported that only one of seven patients with PBC-AIH treated with UDCA monotherapy (15 mg kg^−1^ per day) achieved complete biochemical and histological remission. These findings have caused some scholars to believe that the treatment of PBC-AIH with UDCA is less effective than the treatment of PBC with UDCA and have suggested a sequential therapy involving initial prednisolone (0.5 mg kg^−1^ per day) for two weeks to reduce transaminase and immunoglobulin G (IgG) levels. Other scholars [7–10] believe that treating PBC-AIH with combined prednisolone and UDCA is more likely to improve the biochemical and histological outcomes, to reduce complications, and to improve the patient prognoses than can treatment with one agent alone. Today, the proposition that PBC-AIH can only be effectively treated with UDCA combined with corticosteroid therapy is still controversial [1, 11, 12]. Therefore, we conducted a meta-analysis, with suitable inclusions and exclusions, to evaluate the efficacy and safety of therapies combining UDCA and corticosteroids compared with those of a UDCA monotherapy for PBC-AIH.

## 2. Methods

### 2.1. Study Identification

The relevant studies were identified and selected by searching the databases PubMed, Cochrane Library, EMBASE, CINAHL, and the Science Citation Index (updated to June 2013) [13] with the search terms “ursodeoxycholic acid”, “corticosteroids”, “combination therapy”, “PBC-AIH”, and “randomized controlled trial.” We also carried out a full manual search of all review articles, retrieved original studies, and abstracts.

### 2.2. Inclusion Criteria

The following selection criteria were applied: (i) study design: RCT comparing combination therapy with UDCA/corticosteroids and monotherapy with UDCA; and (ii) study population: patients with PBC with features of AIH identified according to the Paris criteria [4]. PBC-AIH was strictly defined as the association of PBC and AIH. For the diagnosis of each disease, the presence of at least two of the three accepted criteria was required. The criteria for PBC are (1) alkaline phosphatase (AP) levels at least two times higher than the upper limit of normal (ULN) or *γ*-glutamyltranspeptidase (GGT) levels at least five times higher than the ULN; (2) a positive test for antimitochondrial antibodies; and (3) a liver biopsy specimen showing florid bile duct lesions. The criteria for AIH are (1) alanine aminotransferase (ALT) levels at least five times higher than the ULN; (2) serum IgG levels at least two times higher than the ULN, or a positive test for antismooth muscle antibodies; and (3) a liver biopsy showing moderate or severe periportal or periseptal piecemeal lymphocytic necrosis. Duplicated publications were excluded and no language or date limitations were imposed. There was also no limitation on the form of publication.

### 2.3. Data Extraction

The data were independently abstracted from each study by the two researchers (Yan Zhang and Jie Lu) and any disagreement was resolved by consensus. The following data were extracted from each included article: name of the first author, year of publication, number of patients, daily dose of oral therapy, duration of treatment, method used to deal with missing data, liver biochemistry (AP, ALT, aspartate aminotransferase (AST), GGT, IgG, IgM), symptoms, liver histology, death, liver transplantation, death and/or transplantation, and adverse events.

### 2.4. Methodological Quality

The methodological quality of the studies included in the meta-analysis was scored with the Jadad composite scale (Table 1) [14, 15]. This is a five-point quality scale, with low-quality studies having a score of ≤2  and high-quality studies a score of ≥3. Methodological quality was independently assessed by the two authors of this study. Each study was given an overall quality score based on the criteria described above, which was then used to rank the studies. Any disagreement was resolved by consensus.

### 2.5. Statistical Analyses

All analyses were performed with RevMan5.2 (The Nordic Cochrane Centre, The Cochrane Collaboration, 2012). The odds ratio (OR) for each clinical event was presented with its 95% confidence interval (CI). We tested heterogeneity by using the  *χ* ^2^  test and the *I* ^2^ test, and a  *P*  value of <0.10 or an  *I* ^2^  value of >50%  was considered to indicate substantial heterogeneity. A fixed-effects model was used when the heterogeneity test showed a *P* value of >0.10 and an  *I* ^2^  value of <50%; otherwise, a random-effects model was used. We also constructed funnel plots graph to evaluate the presence of publication bias.

## 3. Results

### 3.1. Descriptive and Qualitative Assessments

**Figure 1:**
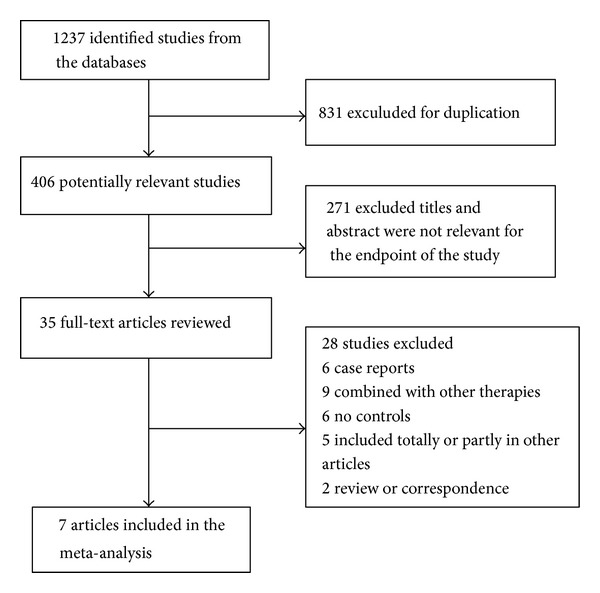
Flow diagram of the studies included in the meta-analysis.

From 1237 studies, we finally selected seven RCTs (Figure 1) [4, 16–21]. They involved 117 patients: 67 were randomized to the UDCA monotherapy groups and 50 to the combination therapy (UDCA and corticosteroid) groups. The baseline characteristics of the seven trials are listed in Table 2. The mean ages ranged from 44 to 55 years and the mean follow-up intervals ranged from 10 to 90 months. The daily dose of UDCA ranged from 10 mg/kg to 15 mg/kg, and the daily dose of corticosteroid ranged from 0.5 mg/kg to 1 mg/kg. The methodological quality scores ranged from 2 to 5 (Table 3). The descriptive results are shown in Table 4.

### 3.2. Evaluation of the Effects of Therapy

The seven RCTs reported the impact of the treatments on the patients' symptoms, but only three studies [17, 18, 20] demonstrated improvement in fatigue, two studies [17, 20] demonstrated improvement in jaundice, and the other RCTs were considered ineffective. All of the included studies agreed that the combination therapy significantly improved liver function and reduced the serum levels of AP and ALT. Liver histology was followed-up in the seven RCTs, and all but one RCT indicated that the combination therapy slowed or even stopped the decline in liver histology [20]. Three studies [4, 19, 21] also reported adverse effects (nausea, vomiting, osteoporosis, aggravated pruritus, and diarrhea), but no serious adverse events occurred.

### 3.3. Meta-Analysis

#### 3.3.1. Pruritus and Jaundice

**Figure 2:**
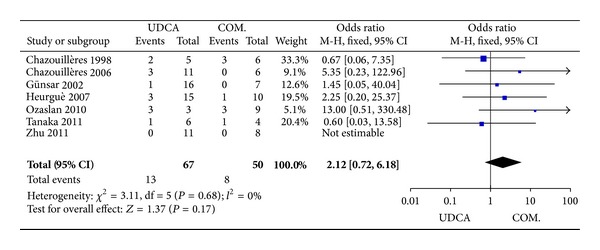
Effects of monotherapy versus combination therapy on pruritus and jaundice in patients with PBC-AIH.

Seven trials [4, 16–21], including 117 patients, reported data regarding these endpoints. The symptoms improved in 13 of 67 patients in the monotherapy groups and in eight of 50 patients in the combination therapy groups. There was no significant heterogeneity (*P* = 0.68, *I* ^2^ = 0%) and no significant differences between the groups (OR 2.12, 95% CI 0.72–6.18, *P* = 0.17; Figure 2).

#### 3.3.2. ALT and AP Levels

**Figure 3:**
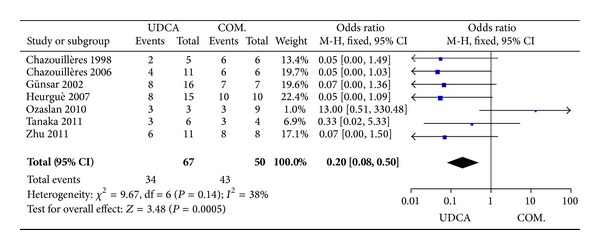
Biochemical parameters of patients treated with monotherapy versus combination therapy for PBC-AIH.

Seven trials, including 117 patients, reported data regarding these endpoints. The symptoms improved in 34 of 67 patients in the monotherapy groups and in 43 of 50 patients in the combination therapy groups. There was no significant heterogeneity (*P* = 0.14, *I* ^2^ = 38%), but there were significant differences between the groups (OR 0.20, 95% CI 0.08–0.50, *P* = 0.0005; Figure 3).

#### 3.3.3. IgG and IgM Levels

**Figure 4:**
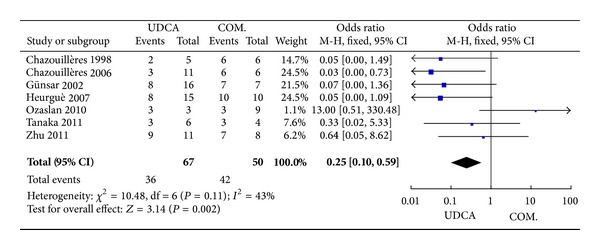
IgG and IgM levels in patients treated with monotherapy versus combination therapy for PBC-AIH.

Seven trials, including 117 patients, reported data regarding these endpoints. The symptoms improved in 36 of 67 patients in the monotherapy groups and in 42 of 50 patients in the combination therapy groups. There was no significant heterogeneity (*P* = 0.11, *I* ^2^ = 43%), but there were significant differences between the groups (OR 0.25, 95% CI 0.10–0.59, *P* = 0.002; Figure 4).

#### 3.3.4. Histological Progression

**Figure 5:**
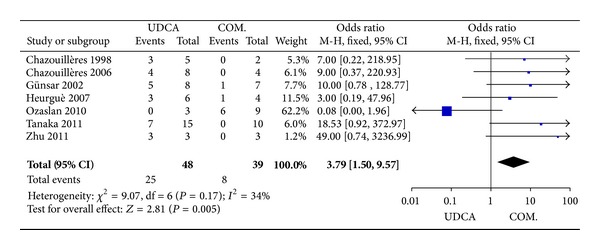
Histological progression in patients treated with monotherapy versus combination therapy for PBC-AIH.

Of the 117 patients (seven trials) who underwent second biopsies, histology declined in 25 of 48 patients in the monotherapy groups and in eight of 39 patients in the combination therapy groups. There was no significant heterogeneity (*P* = 0.17, *I* ^2^ = 34%), but there were significant differences between the groups (OR 3.79, 95% CI 1.50–9.57, *P* = 0.005; Figure 5).

#### 3.3.5. Death

**Figure 6:**
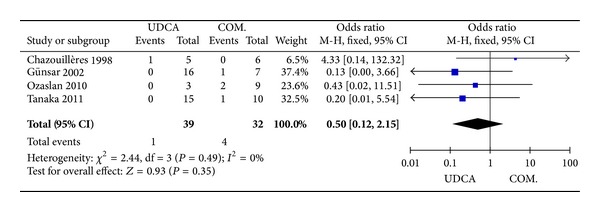
Death in patients treated with monotherapy versus combination therapy for PBC-AIH.

Four trials [4, 17, 20, 21], including 74 patients, reported data regarding this endpoint. The occurrence of death is one of 39 patients in the monotherapy groups and four of 32 patients in the combination therapy groups. There was no significant heterogeneity (*P* = 0.49, *I* ^2^ = 0%) and there were no significant differences between the groups (OR 0.50, 95% CI 0.12–2.15, *P* = 0.35; Figure 6).

#### 3.3.6. Death or Liver Transplantation

**Figure 7:**
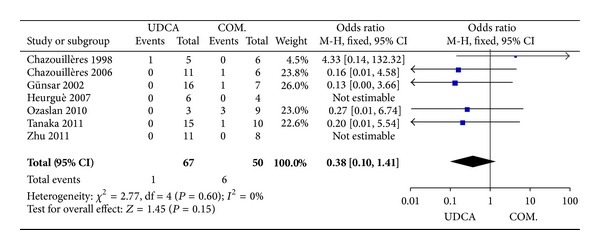
Death or liver transplantation in patients treated with monotherapy versus combination therapy for PBC-AIH.

Seven trials, including 117 patients, reported data regarding this endpoint. It was showen in one of 67 patients in the monotherapy groups and in six of 50 patients in the combination therapy groups. There was no significant heterogeneity (*P* = 0.60, *I* ^2^ = 0%) and there were no significant differences between the groups (OR 0.38, 95% CI 0.10–1.41, *P* = 0.15; Figure 7).

#### 3.3.7. Adverse Events

**Figure 8:**
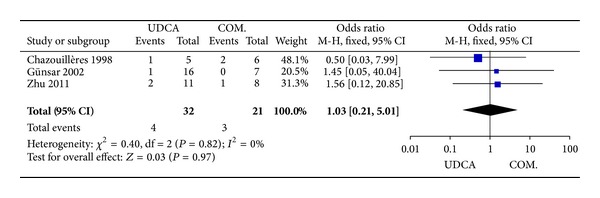
Adverse events in patients treated with monotherapy versus combination therapy for PBC-AIH.

Three trials [4, 19, 21], including 53 patients, reported data regarding this endpoint. The incidence of adverse events are four of 32 patients in the monotherapy groups and three of 21 patients in the combination therapy groups. There was no significant heterogeneity (*P* = 0.82, *I* ^2^ = 0%) and there were no significant differences between the groups (OR 1.03, 95% CI 0.21–5.01, *P* = 0.97; Figure 8).

### 3.4. Sensitivity Analyses

A sensitivity analysis was performed of the six trials in which mid-dose UDCA (mean dose 13–15 mg kg^−1^ per day) was administered. The analysis indicated no differences in clinical events, histological liver changes, or the rate of death/liver transplantation between the UDCA monotherapy groups and the groups receiving a combination therapy of UDCA and corticosteroid. Only one study was a low-quality study (Jadad score ≤ 2). Thus, the meta-analytical results did not change after the exclusion of this study. A period of one year is commonly considered to be too short to evaluate the survival of PBC-AIH patients. Therefore, another sensitivity analysis was performed, including only those studies of long duration (≥24 months). Two trials [4, 19] were excluded because their treatment regimes were too short. We found that the meta-analytical results after excluding these studies did not change either.

### 3.5. Publication Bias

**Figure 9:**
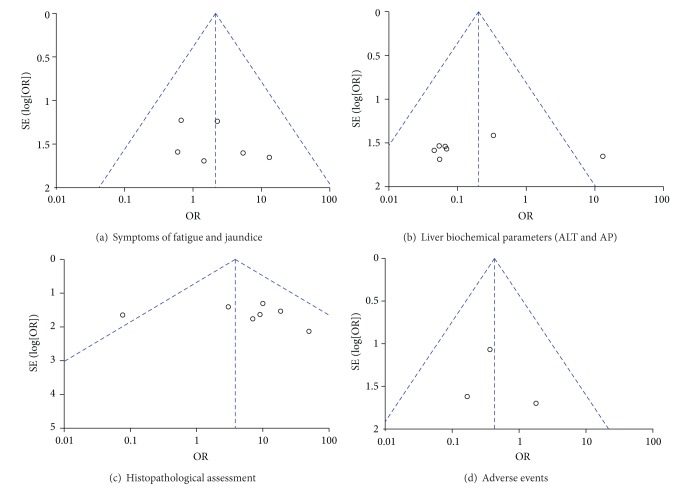
Funnel plots for the meta-analysis.

Figure 9 shows the funnel plots of the meta-analysis. The funnel plots for clinical events showed slight asymmetry, suggesting possible publication bias.

## 4. Discussion

The pathogenesis of PBC with features of AIH is unclear [22], but it is generally agreed that genetic factors, including (HLA)-DR5, may protect against this disease. Because of the complexity and variability of the disease, its treatment remains a major problem for the clinician. There have been many case reports [23, 24] and many data published regarding the clinical features, laboratory features, and pathological characteristics of this disease. Chazouillères et al. [4] reported the results of a 7.5-year followup of 17 noncirrhotic patients with PBC-AIH, which is considered the most influential study on the use of UDCA combined with corticosteroids to treat the disease. They believed that a combination therapy with UDCA and corticosteroids is an ideal treatment for PBC-AIH. According to a retrospective study of a large cohort of patients with PBC-AIH by Ozaslan et al. [25], UDCA alone did not produce a biochemical response in most patients with severe interface hepatitis; these patients require additional therapy with immunosuppression. They concluded that second-line immunosuppressive agents are effective in controlling disease activity in patients that do not respond to conventional immunosuppression. However, the possibility that long-term treatment may cause hormone dependence, resistance, or treatment failure warrants careful consideration [26, 27]. There is no recognized treatment for patients who do not respond to this treatment.

### 4.1. Quality Evaluation of the Studies Included

In this study, we applied stringent inclusion criteria so that the selected studies would have a tight design, good homogeneity, and high credibility. There were no significant differences in the baseline characteristics of the patients in any of the selected studies (e.g., age, sex, race, and serological markers) and little selection bias. However, our systematic review included only studies published in English or any unpublished studies (such as symposium conference records, conference papers, and literature-based evidence from nontraditional sources), which may have led to language bias and publication bias. The funnel plot analyses of symptoms, liver biochemistry, and histopathology showed asymmetry, indicating that there was a certain publication bias.

### 4.2. Clinical Significance

This study has shown that the combination therapy did not differ significantly from the monotherapy in improving fatigue, jaundice, mortality, death/liver transplantation, or adverse events, but was significantly superior to the monotherapy in reducing serum AP, ALT, and other biochemical liver markers. This may be attributable to the effects of corticosteroids in reducing cell edema and relieving the inflammation of the bile duct cells and liver cells [28]. It may also be related to the prevention by UDCA of the increasing permeability of the liver cell membrane, the protection of the liver cell membrane from hydrophobic cytotoxic destruction by bile salts, the inhibition of Kupffer cell activation, and the release of free oxygen radicals, thus protecting the liver cells from damage by oxygen, and thereby improving transaminase levels and the biochemical markers of cholestasis [29, 30]. The literature evaluated was biased because too few studies were included and some were of low quality, so more high-quality studies are required to confirm the conclusions drawn here.

### 4.3. Adverse Effects of Treatment

Three of the included RCTs [4, 19, 21] reported adverse events, whereas the other four did not. These adverse events included osteoporosis, bleeding, aggravated itching, and diarrhea [31, 32]. From a drug safety perspective, the differences in the rates of adverse events between the combination therapy and the monotherapy were not significant (OR = 0.02, 95% CI −0.18–0.21, *P* = 0.87). In clinical trials, treatment efficacy and adverse events should be emphasized equally. If the adverse effects of a therapy are greater than its efficacy, the therapy has no value in clinical applications, regardless of its efficacy.

### 4.4. Limitations of This Study

(1) The number of studies included in this analysis was small. Furthermore, allocation concealment and blinding will have affected the results [32]. Studies [33] have shown that a small allocation concealment sample and no blinding can exaggerate the effects of the intervention by 49% and 52%. Therefore, a rational design must be established before a clinical trial is undertaken to ensure that reliable conclusions are drawn.

(2) Although this research utilized important markers, including mortality, liver transplantation, symptom improvement, and biochemical indicators, quality of life is an equally important indicator. An improvement in the patient's quality of life can determine whether the treatment is truly effective. The Cochrane Collaboration values quality of life as the major measure of treatment efficacy. However, none of the seven papers included in this study measured quality of life. Future studies should consider quality of life as an important indicator of treatment efficacy.

In summary, we recommend that patients diagnosed with the presymptomatic or symptomatic stages of PBC with features of AIH undertake early therapy combining UDCA and corticosteroids, even though there is currently no cure for the disease. This therapy is safe and effective for these patients and can improve their liver biochemistry indicators. Extended treatment may improve the pathological status of the liver, thereby delaying disease progression and improving the patient's quality of life, prolonging his/her life, and reducing the burden on the patient. During treatment with corticosteroids, any opportunity to reduce the dose should be taken, and close observation of the adverse effects of corticosteroids is required, including bleeding, fractures, high blood sugar, high blood pressure, high cholesterol, pancytopenia, and severe infections. Naloxone can be given for itching. Proton pump inhibitors can cure acid reflux and can also prevent stress ulcer bleeding. Oral calcium and vitamin D supplementation can prevent osteoporosis. We suggest that an animal model of autoimmune liver disease should be established and improved in the near future to facilitate research into the pathogenesis of autoimmune liver diseases and target therapies [34, 35]. The development of more specific and more sensitive immunological parameters and genetic diagnostic techniques for the early diagnosis and prognostic evaluation of PBC-AIH is also required. New, more specific, and efficient drugs and treatment programs with fewer adverse effects are also needed.

## Figures and Tables

**Table 1:** Criteria used to grade the quality of RCTs: the Jadad scores.

	Each study was given one point for each “yes” and 0 points for each “no” in response to each of the following questions.
	(1) Was the study described as randomized using the words “randomly”, “random”, or “randomization”?
	(a) An additional point was given if the method of randomization was described and was appropriate (e.g., table of random numbers, computer generated).
	(b) A point was deducted if the method of randomization was inappropriate (e.g., patients allocated alternately, by birth date, or by hospital number).
	(2) Was the study described as “double blind”?
	(a) A point was given if the method of blinding was described and it was appropriate (e.g., identical placebo).
	(b) An additional point was deducted if the method of blinding was inappropriate (e.g., comparing placebo tablet with injection).
	(3) Was there a description of the patients who withdrew or dropped out?
	The maximum number of points was 5.

**Table 2:** Baseline characteristics of the trials included in the meta-analysis.

Authors	Mean age (years)	Monotherapy (*n*)	Combination therapy (*n*)	UDCA dose (mg·kg^−1^·d^−1^)	Corticosteroids dose (mg·kg^−1^·d^−1^)	Duration of treatment	Publication type
Chazouillères et al. [4]	50	5	6	13–15	0.5	23 m	Full text
Günsar et al. [21]	44	13	7	13	0.5	28 m	Full text
Chazouillères et al. [16]	41	11	6	13–15	0.5	90 m	Full text
Heurgué et al. [18]	44	9	4	11–14.7	0.5–1	60 m	Full text
Ozaslan et al. [20]	44	3	9	13–15	0.5	31 m	Full text
Tanaka et al. [17]	54	15	10	10	0.5	73 m	Full text
Zhu et al. [19]	50	11	8	13–15	0.5–1	10 m	Full text

**Table 3:** Jadad quality scores of the trials included in the meta-analysis.

Study	Randomization method	Double blinding	Withdrawals dropouts	Total
Chazouillères et al. [4]	2	2	1	5
Günsar et al. [21]	1	2	1	4
Chazouillères et al. [16]	2	2	1	5
Heurgué et al. [18]	2	1	1	4
Ozaslan et al. [20]	1	2	1	4
Tanaka et al. [17]	1	1	0	2
Zhu et al. [19]	2	2	1	5

**Table 4:** Descriptive results of the randomized trials.

Authors	Symptoms improved		Liver-biochemistry improved		Histology progression		Death		Death or liver transplantation		Adverse events	
	UDCA	COM.	UDCA	COM.	UDCA	COM.	UDCA	COM.	UDCA	COM.	UDCA	COM.
Chazouillères et al. [4]	2/5	3/6	2/5	6/6	3/5	0/2	1/5	0/6	1/5	0/6	1/5	2/6
Günsar et al. [21]	1/16	0/7	8/16	7/7	5/8	1/7	0/16	1/7	0/16	1/7	1/16	0/7
Chazouillères et al. [16]	3/11	0/6	4/11	6/6	4/8	0/4	NR	NR	0/11	1/6	NR	NR
Heurguè et al. [18]	1/6	1/4	3/6	3/4	3/6	1/4	NR	NR	0/6	0/4	NR	NR
Ozaslan et al. [20]	3/3	3/9	3/3	3/9	0/3	6/9	0/3	2/9	0/3	3/9	NR	NR
Tanaka et al. [17]	3/15	1/10	8/15	10/10	7/15	0/10	0/15	1/10	0/15	1/10	NR	NR
Zhu et al. [19]	0/11	0/8	6/11	8/8	3/3	0/3	NR	NR	0/11	0/8	2/11	1/8

UDCA: monotherapy with ursodeoxycholic acid; COM: combination therapy with UDCA and corticosteroids; NR: not reported.
